# Estimating the Size of Key Populations in Kampala, Uganda: 3-Source Capture-Recapture Study

**DOI:** 10.2196/12118

**Published:** 2019-08-12

**Authors:** Reena H Doshi, Kevin Apodaca, Moses Ogwal, Rommel Bain, Ermias Amene, Herbert Kiyingi, George Aluzimbi, Geofrey Musinguzi, David Serwadda, Anne F McIntyre, Wolfgang Hladik

**Affiliations:** 1 Centers for Disease Control and Prevention Center for Global Health Division of Global HIV and TB Atlanta, GA United States; 2 Centers for Disease Control and Prevention Epidemic Intelligence Service Atlanta, GA United States; 3 Public Health Institute Oakland, CA United States; 4 Makerere University School of Public Health Kampala Uganda; 5 Centers for Disease Control and Prevention Division of Global HIV and TB Entebbe Uganda

**Keywords:** sex workers, men who have sex with men, substance-related disorders, HIV, population size

## Abstract

**Background:**

Key populations, including people who inject drugs (PWID), men who have sex with men (MSM), and female sex workers (FSW), are disproportionately affected by the HIV epidemic. Understanding the magnitude of, and informing the public health response to, the HIV epidemic among these populations requires accurate size estimates. However, low social visibility poses challenges to these efforts.

**Objective:**

The objective of this study was to derive population size estimates of PWID, MSM, and FSW in Kampala using capture-recapture.

**Methods:**

Between June and October 2017, unique objects were distributed to the PWID, MSM, and FSW populations in Kampala. PWID, MSM, and FSW were each sampled during 3 independent captures; unique objects were offered in captures 1 and 2. PWID, MSM, and FSW sampled during captures 2 and 3 were asked if they had received either or both of the distributed objects. All captures were completed 1 week apart. The numbers of PWID, MSM, and FSW receiving one or both objects were determined. Population size estimates were derived using the Lincoln-Petersen method for 2-source capture-recapture (PWID) and Bayesian nonparametric latent-class model for 3-source capture-recapture (MSM and FSW).

**Results:**

We sampled 467 PWID in capture 1 and 450 in capture 2; a total of 54 PWID were captured in both. We sampled 542, 574, and 598 MSM in captures 1, 2, and 3, respectively. There were 70 recaptures between captures 1 and 2, 103 recaptures between captures 2 and 3, and 155 recaptures between captures 1 and 3. There were 57 MSM captured in all 3 captures. We sampled 962, 965, and 1417 FSW in captures 1, 2, and 3, respectively. There were 316 recaptures between captures 1 and 2, 214 recaptures between captures 2 and 3, and 235 recaptures between captures 1 and 3. There were 109 FSW captured in all 3 rounds. The estimated number of PWID was 3892 (3090-5126), the estimated number of MSM was 14,019 (95% credible interval (CI) 4995-40,949), and the estimated number of FSW was 8848 (95% CI 6337-17,470).

**Conclusions:**

Our population size estimates for PWID, MSM, and FSW in Kampala provide critical population denominator data to inform HIV prevention and treatment programs. The 3-source capture-recapture is a feasible method to advance key population size estimation.

## Introduction

Key populations, including people who inject drugs (PWID), men who have sex with men (MSM), and female sex workers (FSW), are disproportionately affected by the HIV epidemic. Compared with the general population, higher prevalences of HIV infection have been documented in these key populations because of high-risk sexual behaviors and injecting drugs [1-3].

Understanding the magnitude of the HIV epidemic among these populations requires accurate population estimates. These estimates inform the scale of prevention and treatment programs and are needed for resource allocation, monitoring, and evaluation of the programs [4,5]. Traditional methods of estimating the size of these populations, such as a census, are challenging because of the marginalized nature of these populations. Criminalization of sexual behaviors and drug use, in addition to human rights abuses, and severe stigma keep these populations socially hidden [6].

Even in Uganda’s generalized epidemic, the high HIV prevalence observed in several studies of key populations suggests that they may account for a substantial number of HIV infections [7-9]. In Kampala, HIV prevalence among MSM was estimated at 13.7% and 31.3% among FSW in 2012 [8]. Studies among PWID have estimated the prevalence between 11% and 34% (report by Uganda Harm Reduction Network, 2017). Size estimates for PWID, FSW, and MSM in Uganda and Kampala, in particular, are scarce. Existing estimates for FSW and MSM (13,200 [95% confidence interval 10,200-16,200] and 7900 [95% credible interval (CI) 4400-11,300], respectively) in Kampala are outdated (2012) (report by The Crane Survey, 2012). Hospital data on people in drug abuse treatment suggest that there are 10 PWID per 100,000 people (or 70 to 80 people) in Kampala [10]. As a result, HIV services and policies to address population specific needs are likely underestimated and inadequate.

There are many methods to estimate population sizes, each with various strengths and limitations [4]. Capture-recapture (CRC) is one method that has been used in epidemiology [11-13] to estimate the size of key populations [14-17]. This method begins with an initial capture, as individual members of the target population are sampled, tagged, or offered a unique object, and released back into the population. A short period later (days or weeks), a second, independent capture is sampled; during this encounter, individuals will be assessed or asked whether they have been previously tagged, that is, received an object. Additional captures increase the number of data points from which estimates are generated, generally resulting in more stable and robust key population size estimates [5,11].

We used CRC to estimate the sizes of key populations in Kampala, Uganda, in June and October 2017.

## Methods

### Capture-Recapture

The CRC methodology is described in detail elsewhere [11,13,18-20]. Briefly, 4 main assumptions must hold for CRC to produce accurate population estimates: the population is closed; individual captures are independent; the capture history of each target population member is accurate; and the probability of being captured is homogenous [21]. The degree to which these assumptions are violated determine the accuracy of CRC-based population size estimates.

We defined MSM as men, aged 18 years or above, who self-identified as MSM. We defined FSW as women, aged 15 years or above, who reported currently selling sex for money. We defined PWID as people, aged 15 years or above, who reported currently injecting nonprescription or illegal drugs.

Local community-based organizations were consulted to discuss the selected objects (tags) and recommend peer distributors for each target population. Selected objects had no monetary value, were unique (ie, unavailable in Uganda), and differed according to each population. Different objects were used for each capture. Objects were procured in different colors with each color assigned for distribution in a different Division for quality assurance and to get a crude sense of mobility of objects across Kampala. Objects included keychains with bottle openers and lights, bracelets, and compact mirrors with unique phrases or artwork.

Data from mapping exercises and previous FSW and MSM size estimation conducted in 2011 and 2012 in Kampala were used to generate a range of the number of objects to be distributed in each of the 5 administrative Divisions of Kampala. As the PWID population had no prior population estimates, we utilized data from a nongovernmental organization, Community Health Alliance Uganda, that provided an estimated number of PWID hotspots per Division.

### Data Collection

Peer staff were assigned to a particular Division in Kampala and within that Division, to a set of parishes (there were a total of 99 parishes in Kampala). Each parish had only 1 distributor per capture. A total of 2 peer FSW and MSM distributors were assigned to each of the 5 Divisions, whereas 1 PWID distributor was assigned to each Division. To facilitate independence between captures, new MSM and FSW distributors were selected for each capture stage. For PWID, the distributors remained the same, but were rotated and assigned to a new Division. Peer distributors were asked to visit their assigned parishes and to distribute unique objects in areas where the population members work (FSW) or congregate (MSM and PWID). This included public spaces such as streets, bars, clubs, restaurants, and brothels.

Data collection for PWID and MSM CRC was conducted during June 2017 and during October 2017 for FSW. Each capture was set one week apart to minimize the effect of migration in and out of Kampala. All data were collected using ODK Collect on Android smartphones [22]. When a distributor encountered a likely member of the target population, they verified that the individual (1) was a member of the target population and (2) had not been previously approached by another distributor (in a different Division or parish that same week). Target population members were instructed to keep the unique objects with them for the next 2 weeks and not to give it away. Distributors recorded whether the target population member accepted or rejected an object, estimated the age group (15 to 17 years, 18 to 24 years, or >25 years), and documented the global positioning system (GPS) point of the encounter. MSM who appeared to be under the age of 18 years were not approached. No personal identifiers were collected and no compensation was provided. All questions in the ODK Collect program were contingent on the previous question being recorded to limit missing information. Per the protocol, women in the target population who were estimated to be under 18 years of age were referred for specialized support services.

During captures 2 and 3, members of the target population were asked to produce all of the objects they had received. If the approached population member claimed to have received one, but did not have the object with them, they were asked to identify the correct object from a piece of paper with pictures of 10 to 15 different objects (some similar to the real objects, some very different). Distributors recorded the picture the individual identified, but did not reveal whether they were correct or not. Target population members could have received no object, the capture 1 object, the capture 2 object, or both capture 1 and 2 objects. We defined a recapture as an individual who presented the object or was able to identify the correct object from a set of pictures.

### Statistical Analysis

We calculated 3-source CRC (3SCRC) size estimates for all populations and when unable to calculate a 3SCRC, a 2-source CRC (2SCRC) size estimate was calculated in its place. We summarized 2-source capture histories into a 2×2 contingency table where the rows and columns represent the 2 capture occasions. Population size (N) for this group was calculated using the Lincoln-Petersen estimator assuming independence as follows [23]:


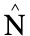
= M_1_*M_2_/R

*where* 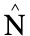 *=* *estimated population size*

M_1_=number of individuals recorded in first capture

M_2_=number of individuals recorded in second capture

R=number of individuals recorded in both captures

As the population size distribution was skewed and violated the normality assumption, we calculated a bootstrap 95% confidence interval for 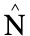
by resampling capture 2 data, 10,000 times (with replacement).

Furthermore, 3SCRC data were aggregated by captures into 2^k^-1 observed frequencies, where k represents the number of captures (k=3). Each capture is listed as either 1 or 0 representing whether individuals are *captured* or *not captured*, respectively. Population sizes and 95% credible intervals (CI) were estimated using a Bayesian approach.

### Latent-Class Bayesian Nonparametric Model

Consider the Kampala FSW and MSM populations to each be a closed finite population of N individuals. We conducted 3 capture stages for each population. Let x_i_=(x_1i_, x_i2_, x_i3_), with x_ik_=1 if the i^th^ individual was captured in the k^th^ FSW capture stage and 0 otherwise. Thus, x_i_,i=1,…,N, represents the complete capture history of each individual in the FSW population (similarly for the MSM population). However, of the N individuals in the population, we are only able to capture n<N, where any individual with a capture history equal to (0,0,0) is unobserved. The total number of unobserved individuals is n_0_=N – n, the number to be estimated. Hence, 3SCRC estimation of the size of a closed population is essentially a missing data problem.

Following Little and Rubin [24], we can use a Bayesian approach to model the mechanism that leads to missing data and then to estimate the number of missing individuals. When there is heterogeneity of capture probabilities among individuals, this modeling is complicated as the heterogeneity induces dependency among capture stages. A mixture model approach is one technique used in CRC to account for individual heterogeneity [25]. Our choice of mixture model is the latent-class Bayesian nonparametric model of Dunson and Xing [26]. This choice of model avoids the model selection problem of choosing an appropriate number of latent classes, K, in advance [26].

The latent-class Bayesian nonparametric model accommodates various forms of capture probabilities and implements an automatic model selection procedure [27]. Uninformative priors, that is, those that have minimal influence on the inference and dominated by the likelihood function, were specified for the Dirichlet process parameter (α~Gamma (0.25, 0.25)) as suggested in the literature [28]. We used K=10 latent classes; 150,000 samples from the posterior distribution were drawn with a burn-in of 10,000 iterations and a thinning interval of 100 iterations to specify the MCMC sampling.

We performed a sensitivity analysis to investigate the robustness of the posterior distribution of N to a range of priors for α. Smaller values favor sparse mixtures, whereas large values favor a more complex joint distribution. Convergence of the Markov Chain Monte Carlo sampling was assessed using trace plots and by setting various burn-in periods. Statistical analysis was performed in R (version 3.4.2) [29] and the Bayesian nonparametric latent-class capture-recapture package (LCMCR; sample R code for LCMCR analysis is available in Multimedia Appendix 1) [30].

The study protocol was approved by the human subjects protection board at Makerere University School of Public Health and the Centers for Disease Control and Prevention (CDC).

## Results

### Sampling

#### People Who Inject Drugs

**Figure 1:**
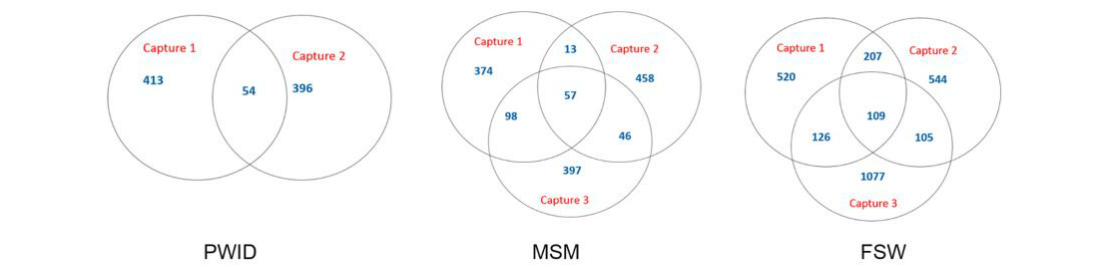
Capture history diagrams for people who inject drugs (PWID), men who have sex with men (MSM), and female sex workers (FSW), Kampala, Uganda, 2017.

Individuals accepted 467 bracelets and refused 5 during capture 1. Individuals accepted 450 bracelets and refused 18 during capture 2. In total, 54 PWID were captured in both capture 1 and 2 (Figure 1). During capture 3, distributors approached and asked 475 PWID to present/identify the object(s); however, recording errors prevented the use of capture 3 data (Figure 1).

#### Men Who Have Sex With Men

Individuals accepted 542 keychains and refused 52 during capture 1. Individuals accepted 574 keychains and refused 26 during capture 2. During capture 3, distributors approached and asked 598 MSM to present/identify the object(s). There were 70 captured in captures 1 and 2, 103 captured in captures 2 and 3, and 155 captured in captures 1 and 3. There were 57 MSM captured in all 3 captures.

#### Female Sex Workers

Individuals accepted 962 mirrors and refused 77 during capture 1. Individuals accepted 965 mirrors and refused 41 during capture 2. During capture 3, distributors approached and asked 1417 FSW to present/identify the object(s). There were 316 captured in captures 1 and 2, 214 captured in captures 2 and 3, and 235 captured in captures 1 and 3. There were 109 FSW captured in all 3 captures.

**Figure 2:**
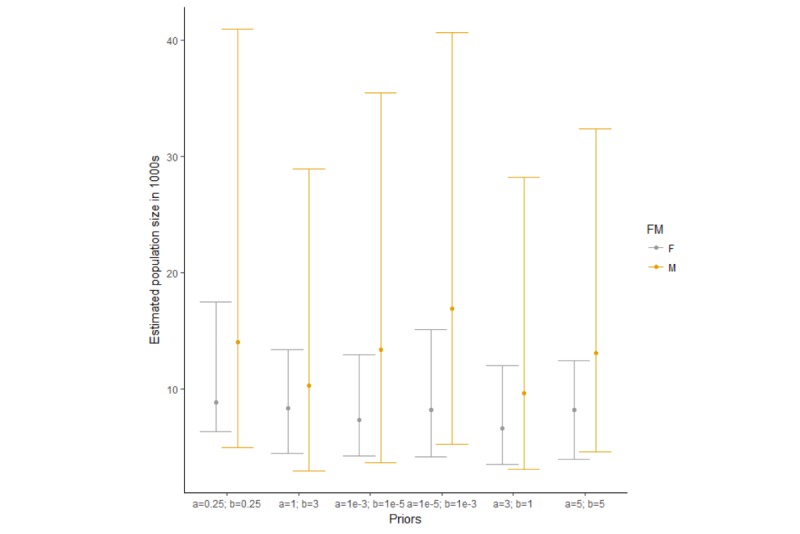
Three-source capture-recapture sensitivity analysis comparing the median and 95% credible intervals of the population size for men who have sex with men and female sex workers, Kampala, Uganda, 2017. Error bars denote the lower and upper bounds of the 95% credible intervals, whereas the filled circles indicate the median. F: female; M: male.

We estimated the number of PWID to be 3892 (95% confidence interval: 3090-5126) using 2SCRC (Table 1). We estimated the population of MSM in Kampala to be 14,019 (95% CI 4995-40,949) and the number of FSW to be 8848 (95% CI 6337-17,470) using the Bayesian approach to 3SCRC. Sensitivity analyses results for the MSM and FSW estimates showed no substantial difference between the estimates as can be seen from the overlapping 95% CI (Figure 2).

**Table 1:** Population size estimates for people who inject drugs, men who have sex with men, and female sex workers, Kampala, Uganda, 2017.

Population^a^	Estimates (95% confidence interval)^b^
People who inject drugs	3892 (3090-5126)
Men who have sex with men	14,019 (4995-40,949)
Female sex workers	8848 (6337-17,470)

^a^Population of people who inject drugs was estimated using 2-source capture-recapture; populations of men who have sex with men and female sex workers were estimated using 3-source capture-recapture with the Bayesian nonparametric latent-class capture-recapture (LCMCR) package.

^b^95% confidence interval for people who inject drugs and 95% credible interval for men who have sex with men and female sex workers.

## Discussion

These population size estimates represent the first use of 3SCRC for FSW and MSM and the first use of 2SCRC for PWID in Kampala, Uganda.

Our 2SCRC PWID estimate of 3892 (95% confidence interval: 3090-5126) is substantially higher than previous estimates (10 PWID per 100,000 people or 70 to 80 PWID total) which were based on clinic data and counts at hotspots [10]. Although we were unable to use data from a third capture of PWID because of data collection errors, our 2-source estimate suggests that injection drug use may be more prevalent than originally expected [10].

We compared our MSM and FSW results with the population size estimates from 2012. Our MSM estimate (14,019) is higher than the 2012 estimate of 7900 (report by The Crane Survey, 2012). Our crude Kampala MSM estimate represents approximately 4% of the adult male population (aged 18 years or above) in Kampala [31]. Factors influencing our MSM population size estimate might reflect long-term migration patterns among MSM, both rural to urban, as well as migration from smaller to larger urban settings [32,33]. To some extent, MSM size estimates are also determined by the proportion of gay men who may or may not practice their same sex sexuality, a potentially large factor given the highly stigmatized climate in Uganda and its accompanying social norms (marriage and having children). Global estimates suggest that MSM represent 3% to 5% of the general population (ie, before taking migration patterns into account) and can be higher depending on the region [34]. To the extent that rural gay men may be more likely to migrate to urban settings compared with rural heterosexual men, we can expect correspondingly larger proportions of adult urban MSM. Furthermore, Kampala and other urban settings are rapidly growing in total general population size; hence, we can expect the use of equal proportional estimates in 2012 and 2017 to lead to higher absolute estimates in 2017.

The FSW result (8848) is lower than the 2012 estimate of 13,200 (report by The Crane Survey, 2012). Our FSW estimate represents approximately 2% of the Kampala adult female population (aged 15 years and above) [31]. Global FSW population size estimates differ depending upon the sampling method and location. In sub-Saharan African FSW estimates generally fall within 0.7% to 4.3% of the adult female population [35].

Both the MSM and FSW 2012 estimates fall within our 95% CI; however, the CRC methods reported here differ from the previous round of population size estimation for MSM and FSW. Although both employed CRC methodology, the previous estimates used respondent-driven sampling (RDS) surveys as capture 2. There was a difference of 9 to 12 months between captures, compared with only a week for our study. A longer period between captures allows for more in- and out-migration (to and from Kampala), violating one of the four assumptions. As a result, fewer recaptures can be expected, resulting in an inflated size estimate.

There were a number of limitations to the design of the estimation activity. Possible violations of the underlying CRC assumptions could influence the validity of our outcomes and may have resulted in inaccurate population sizes and wider confidence intervals. First, we used unique objects as a tagging mechanism to maintain the anonymity of sampled populations. However, not all individuals were carrying the unique object during subsequent captures, complicating the identification of recaptures. In addition, we must assume that the person presenting the object is the person who received the object (an inherent limitation present in anonymous sampling-based CRC). We tried to mitigate the bias involved in tagging individuals with objects by offering individuals the opportunity to identify the objects from a set of pictures, in addition to reducing the time between captures to 1 week. The short time between each capture also gave us more confidence in the assumption of a closed population. Although we recognize that these populations are mobile, there was likely little change over a 1-week period.

To minimize dependencies between captures, we used different distributors for each capture. Nevertheless, the capture probabilities were likely heterogeneous and target population members tagged in capture 1 may have been more likely to be tagged in captures 2 and 3. This is especially true for MSM and PWID, where we collected captures at known MSM- or PWID-friendly venues. Individuals with higher social visibility are more likely captured at these known sites. Individuals with higher social visibility are more likely to be captured, thus our results are likely to be underestimates for all populations. One way to capture individuals with lower social visibility would be to use an RDS survey as the third capture; however, the target sample size would need to be achieved quickly to mitigate in- and out-migration. In addition, one might expand captures to various other data sources (not just object distribution) to include service lists, social media or other Web-based sites to reach those who might not attend venues.

Our final estimates were based on a Bayesian approach to accommodate the complex patterns of heterogeneity between captures and aggregation of homogenous strata into latent classes, whereas other statistical approaches make reasonably strong assumptions about the structure of the joint distribution of capture patterns [27]. In contrast, the latent-class Bayesian approach is a model-averaging technique and attempts to estimate the joint distribution directly from the data as much as possible [27]. Our use of the LCMCR method to estimate the size of hard-to-reach human population is innovative as the approach is not originally developed for analyzing epidemiological data such as those obtained from in-person listing of key populations. The unique individual characteristic of these populations contributes to most of the dependence between capture histories; therefore, the nonparametric latent-class model implemented in LCMCR is reasonable to use.

Working with each of the key populations brought on unique challenges and could have resulted in biased population size estimates. Our definition of each key population was sensitive and it is possible that nontarget population members were counted in each capture. We had substantial challenges finding and training MSM for this activity. In addition, the refusal rate for the unique object among MSM was higher than among the other 2 populations who rarely refused the object (8.8% of MSM compared with 1.2% for FSW and 1.0% for PWID). Furthermore, we found at least one problematic distributor in each target population, which may have biased our results. For example, in capture 3, one particular PWID distributor sampled 54 PWID (all found in the same Division) who had received both objects distributed in capture 1 and 2. Allegedly, all 54 PWID had the exact same color objects. As investigators knew which color had been distributed in each Division (a quality assurance mechanism), and the recorded color of the object had been distributed in another Division, it became clear that the data had very likely been fabricated; hence, we decided to exclude capture 3. There were also anecdotal observations of target population members approaching the distributor hoping to get an object, especially among the FSW population. This suggested that the objects may not always have been given out at random and the members of the target populations did not necessarily have an equal chance of being tagged. Increased monitoring and supervisor would likely help mitigate some of these challenges. One of the benefits of using 3SCRC is the ability to partially account for such dependencies by allowing sources to be examined pairwise (interactions) [13].

In conclusion, we generated new size estimates for key populations in Kampala and demonstrated that 3SCRC is a feasible size estimation method. These estimates will provide critical denominators that may serve as a basis for HIV prevention and treatment program planning by HIV coordinating bodies in Uganda. As we move closer to HIV epidemic control, estimating the size of these key populations will be important to examine and document progress.

## Appendix

Multimedia Appendix 1 Sample R code for LCMCR analysis.

## Abbreviations

2SCRC: 2-source capture-recapture
3SCRC: 3-source capture-recapture
CDC: Centers for Disease Control and Prevention
CI: credible interval
CRC: capture-recapture
FSW: female sex workers
LCMCR: Bayesian nonparametric latent-class capture-recapture package
MSM: men who have sex with men
PWID: people who inject drugs
RDS: respondent-driven sampling

## References

[1] Baral S, Beyrer C, Muessig K, Poteat T, Wirtz AL, Decker MR, Sherman SG, Kerrigan D (2012). Burden of HIV among female sex workers in low-income and middle-income countries: a systematic review and meta-analysis. Lancet Infect Dis.

[2] Aceijas C, Stimson GV, Hickman M, Rhodes T, United Nations Reference Group on HIV/AIDS Prevention and Care among IDU in Developing and Transitional Countries (2004). Global overview of injecting drug use and HIV infection among injecting drug users. AIDS.

[3] Beyrer C, Baral SD, van Griensven F, Goodreau SM, Chariyalertsak S, Wirtz AL, Brookmeyer R (2012). Global epidemiology of HIV infection in men who have sex with men. Lancet.

[4] (2011). UNAIDS.

[5] Abdul-Quader AS, Baughman AL, Hladik W (2014). Estimating the size of key populations: current status and future possibilities. Curr Opin HIV AIDS.

[6] (2016). World Health Organization.

[7] Hladik W, Baughman AL, Serwadda D, Tappero JW, Kwezi R, Nakato ND, Barker J (2017). Burden and characteristics of HIV infection among female sex workers in Kampala, Uganda - a respondent-driven sampling survey. BMC Public Health.

[8] Hladik W, Sande E, Berry M, Ganafa S, Kiyingi H, Kusiima J, Hakim A (2017). Men who have sex with men in Kampala, Uganda: results from a bio-behavioral respondent driven sampling survey. AIDS Behav.

[9] Mathers BM, Degenhardt L, Phillips B, Wiessing L, Hickman M, Strathdee SA, Wodak A, Panda S, Tyndall M, Toufik A, Mattick RP, 2007 Reference Group to the UN on HIV and Injecting Drug Use (2008). Global epidemiology of injecting drug use and HIV among people who inject drugs: a systematic review. Lancet.

[10] (2010). World Health Organization.

[11] Hook EB, Regal RR (1995). Capture-recapture methods in epidemiology: methods and limitations. Epidemiol Rev.

[12] Hook EB, Regal RR (1999). Recommendations for presentation and evaluation of capture-recapture estimates in epidemiology. J Clin Epidemiol.

[13] van Hest R (2007). Capture-Recapture Methods in Surveillance of Tuberculosis and Other Infectious Diseases.

[14] Des Jarlais D, Khue PM, Feelemyer J, Arasteh K, Thi Huong D, Thi Hai Oanh K, Thi Giang H, Thi Tuyet Thanh N, Vinh VH, Heckathorn DD, Moles JP, Vallo R, Quillet C, Rapoud D, Michel L, Laureillard D, Hammett T, Nagot N (2018). Using dual capture/recapture studies to estimate the population size of persons who inject drugs (PWID) in the city of Hai Phong, Vietnam. Drug Alcohol Depend.

[15] Karami M, Khazaei S, Poorolajal J, Soltanian A, Sajadipoor M (2017). Estimating the population size of female sex worker population in Tehran, Iran: application of direct capture-recapture method. AIDS Behav.

[16] Ruiz MS, O'Rourke A, Allen ST (2016). Using capture-recapture methods to estimate the population of people who inject drugs in Washington, DC. AIDS Behav.

[17] Vuylsteke B, Sika L, Semdé G, Anoma C, Kacou E, Laga M (2017). Estimating the number of female sex workers in Côte d'Ivoire: results and lessons learned. Trop Med Int Health.

[18] Nanan DJ, White F (1997). Capture-recapture: reconnaissance of a demographic technique in epidemiology. Chronic Dis Can.

[19] Neugebauer R (1984). Application of a capture-recapture method (the Bernoulli census) to historical epidemiology. Am J Epidemiol.

[20] Qiu C, Shi L (1998). [Capture-recapture methods and its uses in epidemiology]. Zhonghua Yu Fang Yi Xue Za Zhi.

[21] Neugebauer R, Wittes J (1994). Voluntary and involuntary capture-recapture samples--problems in the estimation of hidden and elusive populations. Am J Public Health.

[22] Hartung C, Lerer A, Anokwa Y, Tseng C, Brunette W, Borriello G (2010). ICTD.

[23] Wittes JT, Colton T, Sidel VW (1974). Capture-recapture methods for assessing the completeness of case ascertainment when using multiple information sources. J Chronic Dis.

[24] Little RJ, Rubin DB (2002). Statistical Analysis With Missing Data: Second Edition.

[25] McCrea RS, Morgan BJ (2014). Analysis Of Capture-Recapture Data.

[26] Dunson DB, Xing C (2012). Nonparametric Bayes modeling of multivariate categorical data. J Am Stat Assoc.

[27] Manrique-Vallier D (2016). Bayesian population size estimation using Dirichlet process mixtures. Biometrics.

[28] Si Y, Reiter JP (2013). Nonparametric Bayesian multiple imputation for incomplete categorical variables in large-scale assessment surveys. J Educ Behav Stat.

[29] The R Project.

[30] Manrique-Vallier D (2019). R Package Documentation.

[31] (2016). United Nations Statistics Division.

[32] Lieb S, Fallon SJ, Friedman SR, Thompson DR, Gates GJ, Liberti TM, Malow RM (2011). Statewide estimation of racial/ethnic populations of men who have sex with men in the U.S. Public Health Rep.

[33] Lieb S, Thompson DR, Misra S, Gates GJ, Duffus WA, Fallon SJ, Liberti TM, Foust EM, Malow RM, Southern AIDS Coalition MSM Project Team (2009). Estimating populations of men who have sex with men in the southern United States. J Urban Health.

[34] Cáceres C, Konda K, Pecheny M, Chatterjee A, Lyerla R (2006). Estimating the number of men who have sex with men in low and middle income countries. Sex Transm Infect.

[35] Vandepitte J, Lyerla R, Dallabetta G, Crabbé F, Alary M, Buvé A (2006). Estimates of the number of female sex workers in different regions of the world. Sex Transm Infect.

