# Correction: Estimating the Size of Key Populations in Kampala, Uganda: 3-Source Capture-Recapture Study

**DOI:** 10.2196/19893

**Published:** 2020-05-12

**Authors:** Reena H Doshi, Kevin Apodaca, Moses Ogwal, Rommel Bain, Ermias Amene, Herbert Kiyingi, George Aluzimbi, Geofrey Musinguzi, David Serwadda, Anne F McIntyre, Wolfgang Hladik

**Affiliations:** 1 Centers for Disease Control and Prevention Center for Global Health Division of Global HIV and TB Atlanta, GA United States; 2 Centers for Disease Control and Prevention Epidemic Intelligence Service Atlanta, GA United States; 3 Public Health Institute Oakland, CA United States; 4 Makerere University School of Public Health Kampala Uganda; 5 Centers for Disease Control and Prevention Division of Global HIV and TB Entebbe Uganda

The article entitled “Estimating the Size of Key Populations in Kampala, Uganda: 3-Source Capture-Recapture Study” (JMIR Public Health Surveill 2019;5(3):e12118) published with a technical error that was introduced after proofreading.

The inline figure 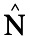 was incorrectly inserted as Figure 4. Figure 4 has now been removed from the manuscript.

The correction will appear in the online version of the paper on the JMIR website on May 12, together with the publication of this correction notice. Because this was made after submission to PubMed, PubMed Central, and other full-text repositories, the corrected article has also been resubmitted to those repositories.

