# Supplementary material for: Estimating the Size of Key Populations in Kampala, Uganda: 3-Source Capture-Recapture Study
Source: JMIR Public Health Surveill. 2019 Aug 12;5(3):e12118. doi: 10.2196/12118 (PMC6771531; doi:10.2196/12118)
Supplement: Multimedia Appendix 1 [file publichealth_v5i3e12118_app1.pdf]

```
# install.packages("Rcapture")
# install.packages("ggplot2")

# Load Libraries

library(Rcapture)                  # model capture recapture data (loglinear model)
library(conting)                   # model capture recapture data-Bayesian
library(ggplot2)                   # graphics
library(reshape)                   # reshaping dataset
library(MASS)
library(CARE1)                     # model capture recapture data (sample coverage)
library(data.table)
library(sjmisc)
library(sjPlot)
library(LCMCR)
library(stargazer)
library(plyr)
library(knitr)
library(kableExtra)
library(dplyr)

library(Rcapture)
library(LCMCR)
library(lattice)
##########################################################################
Read the data exactly
##########################################################################
datafsw<-('
   ch1 ch2 ch3 Fre
      1   0   0 520
      0   1   0 544
      0   0   1 1077
      1   1   0  207
      1   0   1  126
      0   1   1  105
      1   1   1  109')
FSW <- read.table(textConnection(datafsw),header=TRUE)

desc <- descriptive(FSW, dfreq = TRUE)
plot(desc)


##########################################################################
## Fit frequentist loglinear models
##########################################################################
Res <- closedp(FSW, dfreq = TRUE)
print(Res)

##########################################################################
## Heterogeneity graph
```

```
###########################################################################
boxplot(Res)

###########################################################################
## Construct results table
###########################################################################
CI0 <- closedpCI.t(FSW, dfreq = TRUE, m=("M0"))
CIt <- closedpCI.t(FSW, dfreq = TRUE, m=("Mt"))
CIh.c <- closedpCI.t(FSW, dfreq = TRUE, m=("Mh"), h=c("Chao"))
CIh.p <- closedpCI.t(FSW, dfreq = TRUE, m=("Mh"), h=c("Poisson"))
CIh.d <- closedpCI.t(FSW, dfreq = TRUE, m=("Mh"), h=c("Darroch"))
CIh.g <- closedpCI.t(FSW, dfreq = TRUE, m=("Mh"), h=c("Gamma"))
CIth.c <- closedpCI.t(FSW, dfreq = TRUE, m=("Mth"), h=c("Chao"))
CIth.p <- closedpCI.t(FSW, dfreq = TRUE, m=("Mth"), h=c("Poisson"))
CIth.d <- closedpCI.t(FSW, dfreq = TRUE, m=("Mth"), h=c("Darroch"))
CIth.g <- closedpCI.t(FSW, dfreq = TRUE, m=("Mth"), h=c("Gamma") )
Est <- rbind(CI0$CI, CIt$CI, CIh.c$CI, CIh.p$CI, CIh.d$CI, CIh.g$CI,
        CIth.c$CI, CIth.p$CI, CIth.d$CI, CIth.g$CI)[, 1:3]
Est <- cbind(Est, Res$results[1:10, c(3, 5)])
N <- sum(FSW$Freq)
dfR <- N - Res$results[, 4]
CritChisq <- qchisq(0.025, df = dfR, lower.tail = FALSE)
pVal1 <- pchisq(Res$results[, 3], df = dfR, lower.tail = FALSE)[1:10]
Est <- cbind(Est, pVal1)
write.csv(Est, file = file.path(basepath, "Loglinear_results.csv"))
###########################################################################
## ui fit Chi-squared values
###########################################################################
uiChiSq <- (uifit(Res)$fit.stat)[1:10]
pVal2 <- pchisq(uiChiSq, Res$results[1:10, 4], lower.tail = FALSE)
cbind(uiChiSq, pVal2)


###########################################################################
## Bayesian nonparametric latent-class model
###########################################################################

x <- seq(0, 1, by = 0.01)
png(file.path(basepath, "priors.png"))
par(mfrow = c(2,3))
plot(x, dbeta(x, shape1 = 0.25, shape2 = 0.25), type = "l")
plot(x, dbeta(x, shape1 = 1, shape2 = 1), type = "l")
plot(x, dbeta(x, shape1 = 5, shape2 = 5), type = "l")
plot(x, dbeta(x, shape1 = 1, shape2 = 5), type = "l")
plot(x, dbeta(x, shape1 = 5, shape2 = 1), type = "l")
dev.off()
par(mfrow = c(1,1))

FSW[,c(1:3)] <- lapply(FSW[,c(1:3)] , factor)
## nlcm with Jeffrey's hyperprior
```

```
smplr.Jeff <- lcmCR(FSW, tabular = TRUE, K = 10, a_alpha = 0.025,
                    b_alpha = 0.025, seed = 123, buffer_size = 10000,
                    thinning = 100)
post.Jeff <- lcmCR_PostSampl(smplr.Jeff, burnin = 100000,
                             samples = 10000, thinning = 100,
                             output = FALSE)
CI.Jeff <- quantile(post.Jeff, c(0.025, 0.5, 0.975))
CI.Jeff <- as.data.frame(t(CI.Jeff))
CI.Jeff <- CI.Jeff[,c(2,1,3)]
colnames(CI.Jeff)[1:3]<-c("Median","LowerCI", "UpperCI")
CI.Jeff
## nlcm with uniform hyperprior
smplr.unif <- lcmCR(FSW, tabular = TRUE, K = 10, a_alpha = 1,
                    b_alpha = 1, seed = 123, buffer_size = 10000,
                    thinning = 100)
post.unif <- lcmCR_PostSampl(smplr.unif, burnin = 100000,
                             samples = 10000, thinning = 100,
                             output = FALSE)
CI.unif <- quantile(post.unif, c(0.025, 0.5, 0.975))
CI.unif <- as.data.frame(t(CI.unif))
CI.unif <- CI.unif[,c(2,1,3)]
colnames(CI.unif)[1:3]<-c("Median","LowerCI", "UpperCI")
CI.unif
## nlcm with symmetric 5,5 hyperprior
smplr.5_5<- lcmCR(FSW, tabular = TRUE, K = 10, a_alpha = 5,
                    b_alpha = 5, seed = 123, buffer_size = 10000,
                    thinning = 100)
post.5_5 <- lcmCR_PostSampl(smplr.5_5, burnin = 100000,
                             samples = 10000, thinning = 100,
                             output = FALSE)
CI.5_5 <- quantile(post.5_5, c(0.025, 0.5, 0.975))
CI.5_5 <- as.data.frame(t(CI.5_5))
CI.5_5 <- CI.5_5[,c(2,1,3)]
colnames(CI.5_5)[1:3]<-c("Median","LowerCI", "UpperCI")
CI.5_5
## nlcm with positively skewed 1,5 hyperprior
smplr.1_5<- lcmCR(FSW, tabular = TRUE, K = 10, a_alpha = 1,
                    b_alpha = 5, seed = 123, buffer_size = 10000,
                    thinning = 100)
post.1_5 <- lcmCR_PostSampl(smplr.1_5, burnin = 100000,
                             samples = 10000, thinning = 100,
                             output = FALSE)
CI.1_5 <- quantile(post.1_5, c(0.025, 0.5, 0.975))
CI.1_5 <- as.data.frame(t(CI.1_5))
CI.1_5 <- CI.1_5[,c(2,1,3)]
colnames(CI.1_5)[1:3]<-c("Median","LowerCI", "UpperCI")
CI.1_5
## nlcm with negatively skewed 5,1 hyperprior
smplr.5_1<- lcmCR(FSW, tabular = TRUE, K = 10, a_alpha = 5,
                    b_alpha = 1, seed = 123, buffer_size = 10000,
```

```
                    thinning = 100)
post.5_1 <- lcmCR_PostSampl(smplr.5_1, burnin = 100000,
                            samples = 10000, thinning = 100,
                            output = FALSE)
CI.5_1 <- quantile(post.5_1, c(0.025, 0.5, 0.975))
CI.5_1 <- as.data.frame(t(CI.5_1))
CI.5_1 <- CI.5_1[,c(2,1,3)]
colnames(CI.5_1)[1:3]<-c("Median","LowerCI", "UpperCI")
CI.5_1

post <- rbind(data.frame(Prior = rep("Jeffreys", length(post.Jeff)), Size = post.Jeff),
              data.frame(Prior = rep("Uniform", length(post.unif)), Size = post.unif),
              data.frame(Prior = rep("Beta(5,5)", length(post.5_5)), Size = post.5_5),
              data.frame(Prior = rep("Beta(1,5)", length(post.1_5)), Size = post.1_5),
              data.frame(Prior = rep("Beta(5,1)", length(post.5_1)), Size = post.5_1))
post$Prior <- as.factor(post$Prior)
png(file = file.path(basepath, "Prior_sensitivity.png"))
bwplot(Size ~Prior, data = post, layout = c(1,1),
       ylab = "Population size", xlab = "Stick-breaking hyperprior")
dev.off()
ests <- rbind(data.frame(Prior = "Jeffreys", Size = CI.Jeff[1],
                         Lower = CI.Jeff[2], Upper = CI.Jeff[3]),
              data.frame(Prior = "Uniform", Size = CI.unif[1],
                         Lower = CI.unif[2], Upper = CI.unif[3]),
              data.frame(Prior = "Beta(5,5)", Size = CI.5_5[1],
                         Lower = CI.5_5[2], Upper = CI.5_5[3]),
              data.frame(Prior = "Beta(1,5)", Size = CI.1_5[1],
                         Lower = CI.1_5[2], Upper = CI.1_5[3]),
              data.frame(Prior = "Beta(5,1)", Size = CI.5_1[1],
                         Lower = CI.5_1[2], Upper = CI.5_1[3])
              )

write.csv(ests, file = file.path(basepath, "Posterior_summary_by_hyperprior.csv"))
```
